# Quantitative label-free single cell tracking in 3D biomimetic matrices

**DOI:** 10.1038/s41598-017-14458-x

**Published:** 2017-10-26

**Authors:** Jiranuwat Sapudom, Johannes Waschke, Katja Franke, Mario Hlawitschka, Tilo Pompe

**Affiliations:** 1 Institute of Biochemistry, Faculty of Biosciences, Pharmacy and Psychology, Universität Leipzig, Leipzig, 04103 Germany; 2 Institute of Computer Science, Faculty of Mathematics and Computer Science, Universität Leipzig, Leipzig, 04103 Germany; 3 Professorship for Computer Graphics, Faculty of Computer Science, Mathematics and Natural Science, Hochschule für Technik, Wirtschaft und Kultur Leipzig, 04277 Leipzig, Germany

## Abstract

Live cell imaging enables an observation of cell behavior over a period of time and is a growing field in modern cell biology. Quantitative analysis of the spatio-temporal dynamics of heterogeneous cell populations in three-dimensional (3D) microenvironments contributes a better understanding of cell-cell and cell-matrix interactions for many biomedical questions of physiological and pathological processes. However, current live cell imaging and analysis techniques are frequently limited by non-physiological 2D settings. Furthermore, they often rely on cell labelling by fluorescent dyes or expression of fluorescent proteins to enhance contrast of cells, which frequently affects cell viability and behavior of cells. In this work, we present a quantitative, label-free 3D single cell tracking technique using standard bright-field microscopy and affordable computational resources for data analysis. We demonstrate the efficacy of the automated method by studying migratory behavior of a large number of primary human macrophages over long time periods of several days in a biomimetic 3D microenvironment. The new technology provides a highly affordable platform for long-term studies of single cell behavior in 3D settings with minimal cell manipulation and can be implemented for various studies regarding cell-matrix interactions, cell-cell interactions as well as drug screening platform for primary and heterogeneous cell populations.

## Introduction

Cell dynamics, including migration, cell division and cell-cell interaction are fundamental processes in development, tissue repair and disease^1–6^. These processes are specifically modulated by the microstructural as well as biomechanical properties of the extracellular microenvironment^2,7–9^. As *in vivo* studies are frequently limited to short-term, low-resolution investigations, various approaches have been developed to mimic physiologically and pathologically relevant three-dimensional (3D) microenvironments *in vitro*, overcoming non-physiological conditions of standard 2D cell culture setups, too^10–15^. For instance, fibrillar, type I collagen (Coll I) based matrices and synthetic hydrogel materials are increasingly applied as 3D cell culture scaffolds, replicating key features of *in vivo* extracellular matrices (ECM)^12,16–18^.

To study the dynamic cell behavior of heterogeneous cell populations in complex engineered microenvironments in a precise manner, a continuous observation of cells over a period of time, rather than a snapshot at certain time points, is required. Many imaging approaches, e.g. confocal, differential interference contrast, phase contrast microscopies, offer low-invasive and high-throughput spatio-temporal data of cells^6,19–21^. Single cell analysis of those data uses advantages of the respective imaging approach and allows for continuous single cell studies for 2D and 3D cell cultures answering biomedical questions on the impact of microenvironmental parameters on migration, proliferation and differentiation of various cell types. Quantitative image-based analysis is therefore an active field of current life science.

However, the major obstacle of studying single cell behavior at high temporal and spatial resolution using image-based analysis techniques is the lack of an automated quantitative analysis tool, which allows continuous long-term analysis of large number of living cells. Only in that way, statistically relevant results can be revealed and long-term cell fate, like differentiation and cell cycling, can be studied. The underlying problem frequently arises from the low contrast of obtained images from weakly scattering cells. In biomimetic 3D microenvironments this problem is enhanced by overlaid features from contrast-generating microstructures, fibrillar ECM or porous scaffolds. To overcome such a problem, fluorescent microscopy of labelled cells is often used, offering high contrast data, which allows an automated tracking of cells. However, fluorescently labelling (e.g. cell membrane and nucleus staining dyes), or expression of fluorescent proteins in cells (e.g. green fluorescence proteins), as well as the long-term fluorescent illumination for image acquisition induce cell toxicity and phototoxicity as well as changes in cellular behavior^6,22–25^. Moreover, several highly relevant primary cell types are difficult to be labelled *per se* ^23,26^, like hematopoietic stem cells, endothelial cells and macrophages.

Due to the variance of available imaging approaches, various cell detection algorithms to analyze the spatio-temporal microscopy data have been developed in the last decade^27^. Most of cell detection methods are based on the combination of intensity thresholding, edge detection, active contours, template-matching and a large number of customized pre-processing filters. The first image analysis algorithms focusing on 3D single cell tracking used fluorescently labelled cells, with post-processing based on a mix of intensity thresholding and template matching^28^. In order to study cells in a 3D microenvironment with minimal cell manipulation, label-free 3D cell tracking approaches using phase contrast^29^ and bright-field microscopy^30^ were developed to avoid the major concern of phototoxicity. However, the limitation of both above mentioned methods relies on the cell detection based on a correlation of a whole 2D or 3D refraction template of a cell containing characteristic patterns, which requires a complex library of cell templates and a high computational power. Moreover, phase contrast microscopy leads to strong background features in complex biomimetic 3D matrices, like fibrillar collagen type I (Coll I) networks.

Here, we present a new 3D single cell tracking approach, which allows to overcome several problems in the dynamic cell analysis mentioned above. The method is independent from a complex library of cell templates and specific parameters like cell shape, cell intensity and cell types, but still allows a precise cell detection in all three spatial dimensions. It enables cell position detection in z-direction on a sub-pixel resolution level. Using standard bright-field microscopy, our new detection algorithm allows to follow a large number of cells in 3D volumes over time periods of several days, including analysis of migratory characteristics, cell cycling and morphological change in time-dependent manner. Most importantly by using standard bright-field microscopy and new iterative image analysis algorithms it resembles a highly efficient and affordable approach for single cell tracking in biomimetic 3D environments.

## Results and Discussion

**Figure 1:**
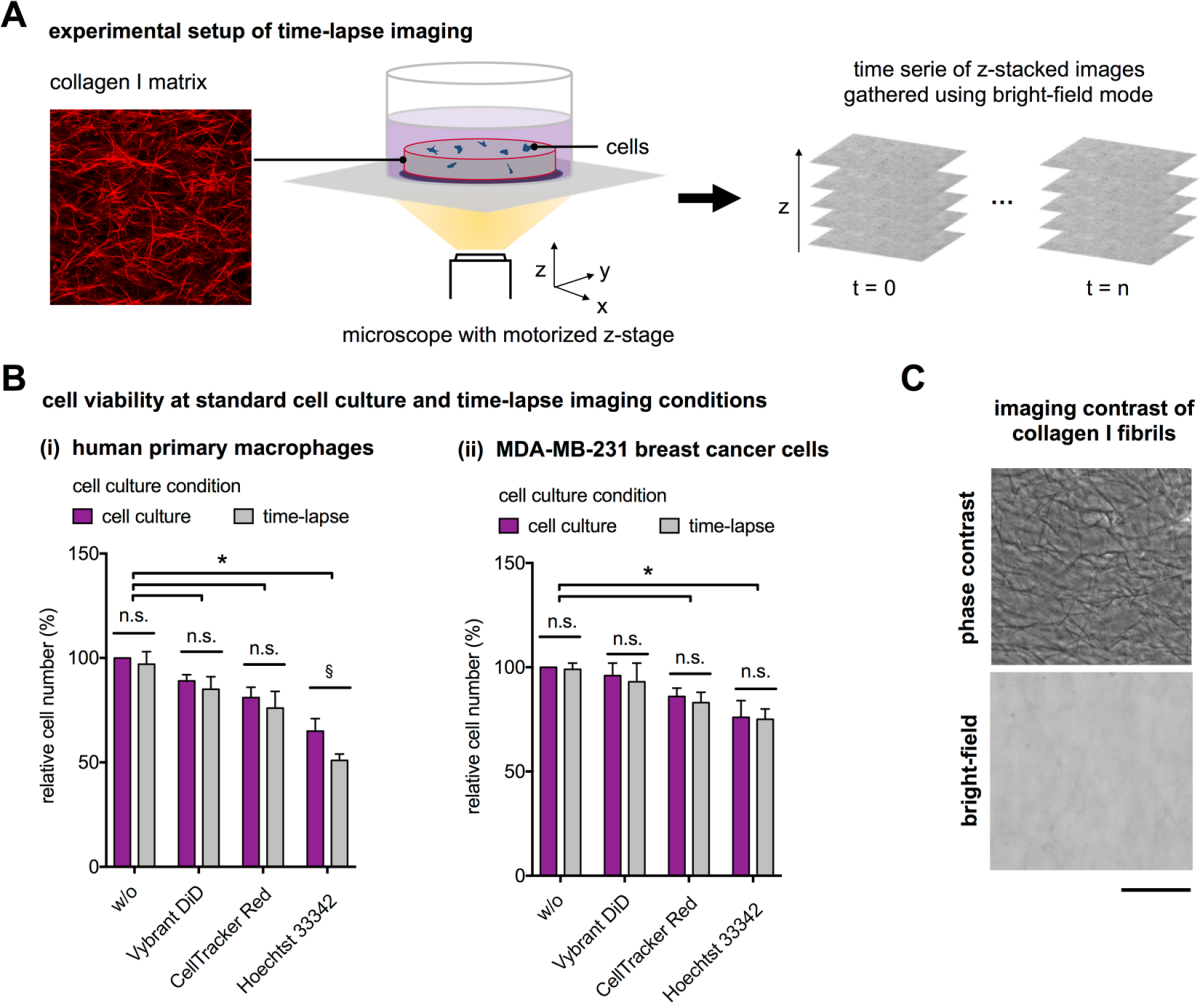
Experimental setup and cell viability tests. (**A**) Schematic illustration of 3D label-free time-lapse imaging using bright-field microscopy in biomimetic Coll I matrices. (**B**) Comparison of cell viability of non-labeled and fluorescently labelled (i) human primary macrophages and (ii) MDA-MB-231 breast cancer cells with Vybrant DiD, CellTracker Red and Hoechst 33342 at standard cell culture condition and time-lapse conditions. Data are shown as mean ± SD. * and § represent statistic significant p < 0.05 for comparison with non-labeled cells, and between two conditions, respectively. (**C**) Optical image signal of Coll I fibrils from phase contrast and bright-field imaging (Scale bar = 20 µm).

To study the dynamic cell behavior, specialized fluorescent staining probes and genetic manipulation by transfection of fluorescent protein labels are usually required, which leads to a decrease of cell viability, as described above. To demonstrate such effects and better motivate our new 3D tracking platform, we firstly studied the impact of commercial fluorescent labelling dyes in cell tracking applications. Based on that, we then describe in detail our new detection approach based on bright-field microscopy and, hence, eliminating cell labeling and phototoxic effect of fluorescent microscopy. Finally, we demonstrate the application of our new technology in a study of migration analysis of primary human macrophages in 3D biomimetic matrices based on fibrillar Coll I. For all experiments, 3D Coll I matrices were reconstituted at the concentration of 2 mg/ml at pH 7.5 resulting in fibrillar networks with average pore size of 10 µm as reported previously^8^. The experimental setup is depicted in Fig. 1A.

### Limitation of fluorescently labelling probes in long-term live cell imaging

To demonstrate the limitation of commercial fluorescence probes in single cell tracking approaches, we tested the effects of routinely used fluorescent dyes, namely Vybrant DiD (cell non-permeate membrane staining), CellTracker Red CMPTX (cell permeate elastase-dependent fluorescent dye) and Hoechst 33342 (cell permeate cell nucleus staining), on cell viability of primary human macrophages and the MDA-MB-231 breast cancer cell line in long-term experiments of 4 days using a commercial WST-1 assay. The cell viability experiments were performed after cultivation both at standard cell culture (standard incubator with 37 °C, 95% humidity, 5% CO_2_) and at time-lapse conditions on a microscope stage in bright-field mode with incubator chamber (37 °C, 95% humidity, 5% CO_2_) and with a low light exposition time of 50 ms per image in xy-plane, 100 images per z-stack and repeated stack acquisition every 10 min. For labelling of cells, recommended concentrations of labelling probes were applied according to the manufacturer’s protocol. As shown in Fig. 1B, primary human macrophages (Fig. 1B(i)) and MDA-MB-231 cells (Fig. 1B(ii)) treated with fluorescently labelling probes demonstrated a significant decrease of cell viability at both conditions. Both cell-permeant probes (Hoechst 3342 and CellTracker Red CMPTX) lead to a higher decrease in cell vitality of both cell types in comparison to the non-permeant, membrane probe (Vybrant DiD), which agrees to previous reports^31,32^.

Although cytoplasmic and nucleus staining are well suited for *in vivo* as well as *in vitro* single cell tracking approaches due to their uniform staining, those probes exhibit a higher cytotoxicity^22^, conflicting non-interfering cell studies. Non-permeant probes are known to non-uniformly staining cell membrane components, which can contribute to biased cell detection^33^. Another disadvantage of fluorescently labelling cells is the bleaching of fluorescent probes. Although we used low intensity bright-field illumination, we also observed label bleaching in our experimental setup after several hours of imaging in dependence on cell type and exposition time. While the latter problem can be decreased by transfection of cells with plasmid to express fluorescent proteins, the transfection process again influences cell phenotype and behavior and is frequently not applicable to many primary cell types^23^. Moreover, one has to keep in mind, that fluorescence microscopy requires in general a higher light intensity than bright-field microscopy leading to even stronger phototoxicity and bleaching effects^23,25^.

By comparing cell viability of non-labelled cells at standard cell culture and time-lapse conditions no significant reduction was observed for both cell types. The results indicate a negligible phototoxicity for the mild conditions in the bright-field microscopy setup.

### Development of a quantitative 3D single cell tracking platform

As discussed in the introduction, there are two computational approaches to track non-labelled cells in 3D matrices. One is based on image data from phase contrast microscopy^29^ and the other using bright-field microscopy^30^. Although phase contrast microscopy provides high contrast of cells, it cannot be applied in 3D fibrillar matrices. As shown in Fig. 1C, phase contrast microscopy provides high contrast of matrix features like Coll I fibrils, limiting a precise detection of cells. Bright-field images do not exhibit high contrasting image details of fibrillar networks (Fig. 1C), but provide low-contrast images of cells at the same time. Hence, cell detection algorithms for bright-field microscopy is more challenging.

Current cell detection methods of bright-field microscopy data rely on 2D or 3D templates based on the refraction signal characteristic of a reference cell. The algorithms calculate the correlation between the obtained templates and any part of the microscope image. If the local correlation is high, the respective image region is allocated as cell^29,30^. The approach based on 2D and 3D templates is applicable for cells of relatively constant size and shape. Generally, cells constantly change their size and shape while migrating^7^. Hence, cell detection using 2D and 3D templates requires complex and versatile templates, as well as a resource-consuming algorithm, since many cell orientations, shapes and sizes must be e\valuated. To circumvent those problems, we developed a new computational framework to study cell behavior in 3D microenvironments from bright-field microscopic images using two 1D templates of z-intensity profiles instead of a tremendously complex library of 2D or 3D templates.

**Figure 2:**
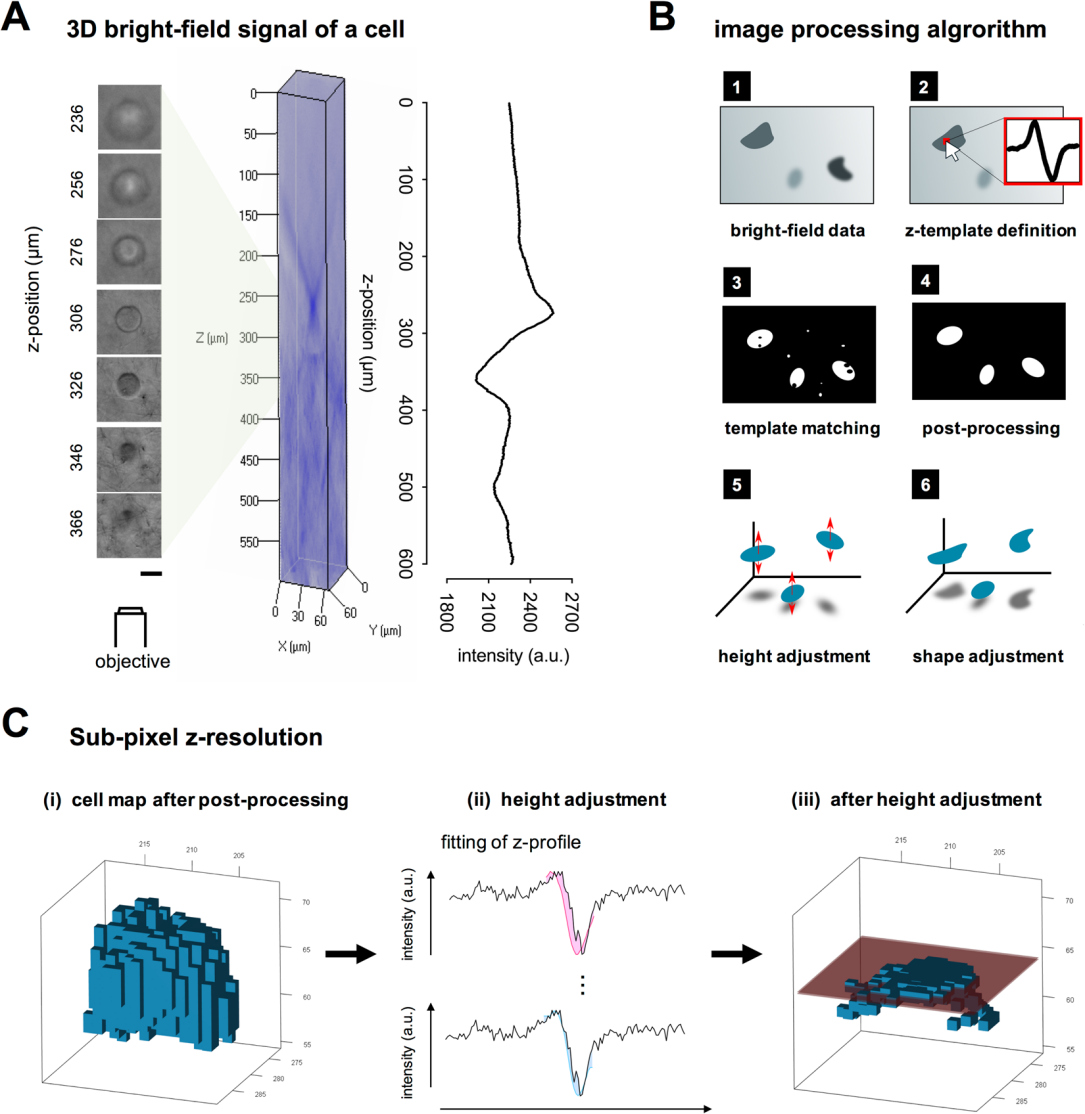
Principle and algorithm of 3D single cell tracking at single cell level. (**A**) Image series of bright-field image and its signal pattern as a function of z-position (left column: Scale bar = 20 µm). (**B**) Image processing algorithm. (**C**) Determination of sub-pixel z-resolution: (i) a cell map after post-processing was used to determine the z-position, (ii) at every voxel, a z-profile (line) was fitted with the cell template until the best matching (minimized filled area) is reached, (iii) a result of sub-pixel z-position determination. The red box visualizes the 95% confidence interval of z-position.

The developed algorithm of cell detection (outlined in Fig. 2B) and trajectory reconstruction subdivides into 4 major steps:

1. cell detection by template matching using a manually selected z-template
2. determination of z-position of each detected cell
3. correction of detected cell shape
4. reconstruction of cell migration trajectories and analysis.

The implementation of these steps is described in detail below.

#### Detection of cell in a 3D volume

The detection of cells in 3D Coll I matrices is based on the bright-field refraction characteristics (Fig. 2A). To detect cells in 3D matrices, a one-dimensional (1D) template of the z-intensity profile was generated by a manual selection of a single cell (*‘cell template’*) and a homogenous, cell-free part of image background (*‘background template’*) in the 3D volume. Supplementary Fig. S3 depicts exemplary templates of a cell and background. Here, we have to mention that the bright-field signal characteristic sensitively depends on the microscope setup (condensor, objective, apertures) and cell type as well. Therefore, a specific template selection for each experiment is an appropriate way to account for the variable intensity characteristics. Next, cells were detected by calculating the Pearson correlation of z-intensity profile at each xy-pixel position of the 3D image volume with the manually selected 1D templates. If the correlation of the z-intensity and the *cell template* was higher than the correlation of the z-intensity and the *background template*, the xy-position is assigned as part of a cell (Fig. S4(ii)). The resulting cell map contained noise pixels, which are caused by the diffraction from Coll I fibers and other artifacts. Hence, a further image processing step to eliminate noise objects was performed by applying an erosion-like filter on the image data, which removed noise based on its density in the cell map (Fig. S4(iii)). For example, an element is removed from the cell map if less than 20% of the neighbors were detected as cells. More technical details on cell detection in the 3D volume are described in the supplementary information in the 3D cell detection section.

Template-based algorithms are among the state-of-the-art methods for object detection with existing applications for cell detection given in the introduction^27,29,30^. However, a main difference of our work compared to existing approaches is, that we use a simple 1D template of the z-intensity signal (Fig. S3). The first advantage of this approach is that cells can be detected regardless of their shape and size. In contrast, 2D or 3D templates would limit detection to objects similar to the template size. Especially when detecting elongated cells, multi-dimensional templates would require a heavily increased search space considering all possible rotations of a cell. The second advantage of our approach is the capability to detect morphological cell characteristics inside the xy-plane in a subsequent step as described below and illustrated in Fig. S5 and S6. Furthermore, the manually selected template could be replaced by the correct theoretical function that describes the optical refraction characteristic of the microscopy setup and the cell. However, details on this option are out of scope of the current work.

A common computational problem in cell detection arises when multiple cells are very close to each other and their refraction patterns interfere. This phenomenon can occur when cells are situated closer than 50 µm in z-direction. As we are currently interested to detect sparse cells in an open matrix volume only, we did not investigate technical workarounds for this problem.

#### Determination of vertical (z-) position

The major problem to precisely determine the z-position is the inherent lower resolution of optical microscopy in z-direction in comparison to the xy-plane, roughly by a factor of 10. We show herein, that we were able to handle this challenge using the z-intensity template fitting resulting in similar resolution in xy- and z-direction without increasing the technical complexity of the microscope setup.

In our microscope setup we used a 5.75 times lower resolution in z-direction than in xy-plane (10 × objective with NA 0.3; xyz voxel sizes is 0.87 µm × 0.87 µm × 5 µm). For each voxel of a detected cell (Fig. 2C(i)), a curve fit between the 1D z-intensity cell template and the actual z-intensity profile was calculated (Fig. 2C(ii)). This fit involves translation along the z-axis and limited linear stretching. Its purpose is to make our technique resilient towards minor profile variations caused by sampling artifacts and noise in microscope images. We derive the z-position from the position of the best fit of our cell template along the z-axis (Fig. 2C(iii)). This step is repeated to calculate the z-position for multiple voxels of a cell. The z-position information of 50% of the central (in xy-plane) voxels is used to determine a precise z-coordinate for the cell as a whole. Voxels representing the cell border are ignored since the z-signal is weak and affected by the almost parallel orientation of the cell border along the z-direction, which would lead to a wrong z-position calculation. Besides the exact cell z-position, a 95% confidence interval of the cell z-position can be derived, indicating a z-position determination of the cell with sub-voxel precision in the range of 0.5 µm (Fig. 2C). This number is similar to the resolution of the xy-plane along us the detect cell positions in 3D volumes with similar resolution in all three spatial dimensions, a result which is important to reliably cell migration characteristics in 3D.

#### Correction of detected cell shape

Up to that step, cell voxels with high similarity of their z-intensity profile to the cell template were detected. Border regions of cells were ignored by the detection algorithm to eliminate problems from the weak refraction profile, see above. To precisely detect the full cell shape, a post-processing step was applied to fill the full cell shape. A common method to restore cell shape is 2D region growing, which expands a cell to neighboring regions with similar intensity. This method works well for cells with a distinct color compared to the background region, which is not the case for our data. We solve this problem by extending the region growing algorithm. Instead of using only the color value of one image layer, we use again a correlation of the z-intensity profiles as a stopping criterion for the growing process (see also Supplementary Video 1). The basic principle is to enlarge the cell area along the xy plane as far as the local z-intensity profile has a high Pearson correlation coefficient in comparison to the z-intensity profile at the center position of the respective cell. The result is better than the original cell detection step, since we do not use the general reference *cell template*, but we compare to the z-intensity profile of the center of the currently processed cell. Using this algorithm, cell shapes could be reliably detected in xy-plane as show in Fig. S5 (for macrophages as a representative of round cells). Based on the cell shape, it is possible to calculate characteristic parameters like cell area, roundness, aspect ratio and cell body orientation (see supplement information in cell shape parameters section).

We additionally challenged the algorithm by another dataset of elongated breast cancer cells (MDA-MB-231) characterized by a mesenchymal migration mode in the 3D matrices. As shown in Fig. S6 even the morphology of elongated cells can be nicely detected. As the z-intensity profile of very thin and elongated filopodias of cells, similar to the feature size of the collagen fibrils of the 3D scaffolds, does not correlate to the *cell template* such cellular structures are not detected. Hence, such very thin features have to be considered as the limit of the algorithm as demonstrated in Fig. S7 for cells of different morphology.

#### Reconstruction of cell migration trajectories from time series of 3D volumes

As the last step of our single cell tracking algorithm, nearest neighbor detection was implemented as a common technique for the reconstruction of single cell trajectories (Fig. S3)^27,34^ in time series of 3D volumes. Nearest neighbor methods are sufficient to reconstruct cell trajectories if the time span between two consecutive time steps is small enough, so that overlapping cell area or a small migrating distance are guaranteed. However, nearest neighbor tracking fails when the migrated distance between two time steps is larger than the distance between the cell and any of its neighbors. In such cases, wrong decisions are made and tracking possibly continues with the wrong cell. In our setup, we assured sufficient reliability of nearest neighbor detection by using time steps of 10 to 15 min, which was enough to track the investigated cell types in 3D. However, for faster migration processes of other cell types or microenvironments these time steps can be adapted down to 2 min for our microscope setup. Additionally, our cell tracking platform offers a manual track correction in case of problems and errors in automated tracking algorithms. Too low image frequency, high local cell densities, as well as optical artifacts from the 3D matrix might disturb automated tracking and require trajectories to be corrected. The module for trajectory reconstruction in our developed toolbox can be extend using other algorithms, e.g. Kalman filter or a combination of nearest neighbor methods and cell shape parameters.

### Application of the 3D single cell tracking platform

**Figure 3:**
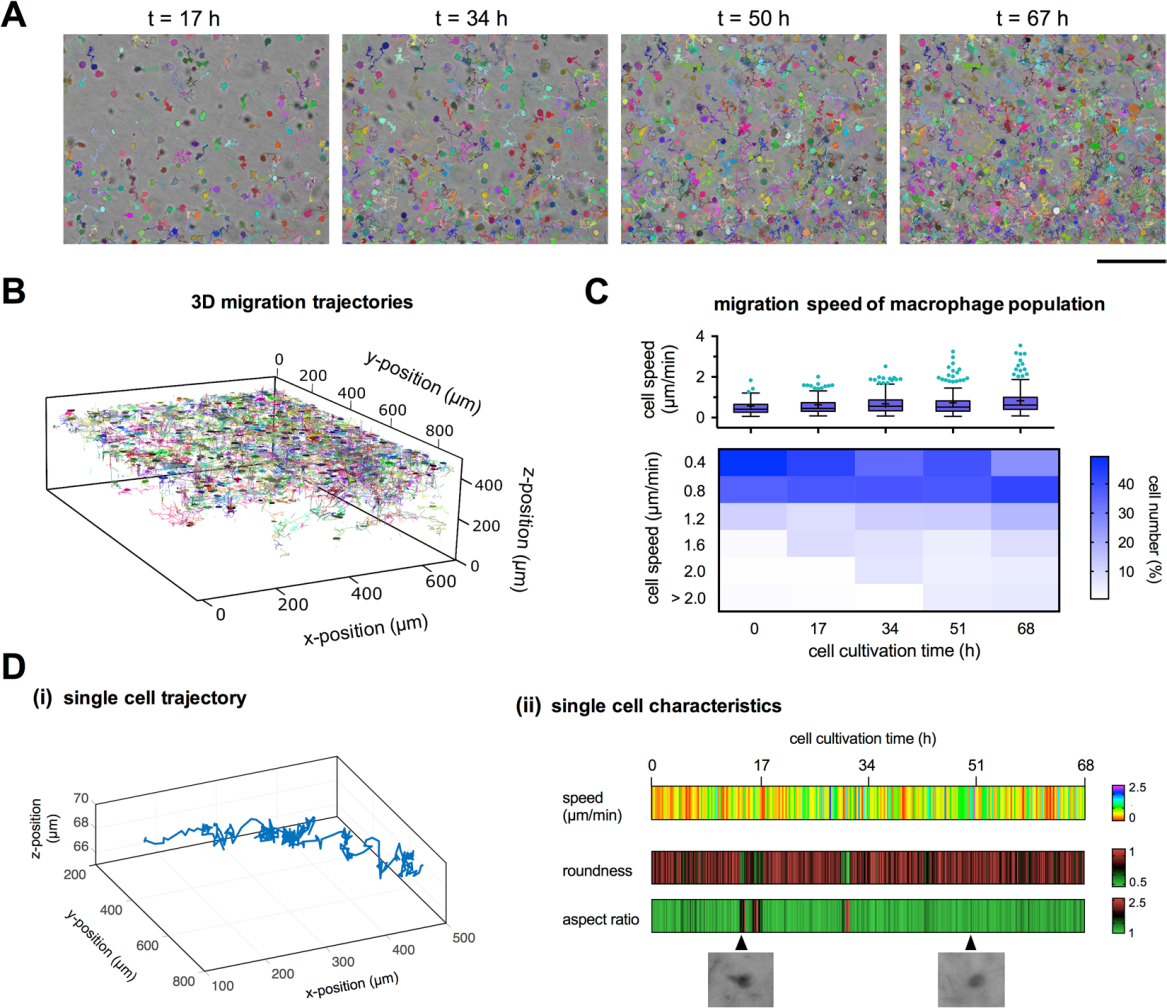
Implementation and quantitative analysis of label-free single cell tracking of human primary macrophages in 3D Coll I matrices. (**A**) Cell migration trajectories at 17 h, 34 h, 50 h and 67 h time points. (Scale bar = 200 µm). (**B**) Visualization of cell migration trajectories in 3D. (**C**) Quantitative population analysis of mean cell migration speed distribution at all and different time points. (**D**) Qualitative analysis of cell migration at single cell level over 67 h cultivation time. (i) 3D visualization of single cell trajectory and (ii) single cell migration speed and morphological parameters such as roundness and aspect ratio.

In the last section, we present an example, where we applied our newly developed method on a time-lapse dataset of primary human macrophages cultivated on 2 mg/ml Coll I matrices, a biomaterials system to nicely mimic 3D cellular microenvironments of various physiological and pathological situations *in vitro* ^35^. The data used in this experiment account for around 125 GB and contained 125 z-stacked images (xy-plane: 1388 × 1040 pixels; z-interval: 5 µm; total 3D volume (x × y × z): 1200 µm × 900 µm × 625 µm) and 408 time steps (image acquisition at 10 min intervals for 68 h), which corresponds to a total of 51000 images. Using a computer with Intel® Core™ i7 CPU 3.40 GHz and 32 GB ram, cell detection of the whole dataset took approximately 3 h and trajectory reconstruction took approximately 5 min, which demonstrates the efficacy of our tracking algorithm. Furthermore, the high efficacy potentially enables real-time analysis in our microscope system. Figure 3A and Supplementary Video 2 illustrates the analysis of the time series dataset using the developed techniques. The percentage of complete tracks versus tracks that need manual correction are roughly 80% for the provided example of 173 macrophages, which we believe is quite efficient. The time for manual correction depends on cell type, image quality and segmentation results. It takes around 3–4 hours for a large dataset of an investigated sample volume and cell number as provided in our example. Reconstructed single cell trajectories of 173 cells are shown in Fig. 3B.

The outputs of our single cell tracking approach, like trajectories and time-resolved cell morphology, provide insight into cell plasticity and migratory phenotypes. The resulting data can be used to answer scientific questions concerning both single cell behavior and cell populations in engineered 3D cellular microenvironments. The quantitative population analysis of mean cell migration speed of 173 macrophages over the whole duration of the experiment of 68 h is shown in Fig. 3C, demonstrated an increase of mean migration speeds of the cell population from 0.6 ± 0.9 µm/min to 0.8 ± 0.7 µm/min (mean ± SD) at time point 0 h and 68 h, respectively. A closer look on the speed distribution reveals that the fraction of cells with low migration speed (<0.5 µm/min) was decreased (from 49% to 26% at time point 0 h and 68 h, respectively) and the fraction of cells with high migration speed (>2 µm/min) was increased (from 1% to 6% at time point 0 h and 68 h, respectively) as a function of cell cultivation time. From the high variance of migration speed of the different cells, it is apparent that meaningful and statistically relevant data from heterogeneous primary cell populations can only be revealed when a high cell number is analyzed, stressing an implementation of our developed platform for automated tracking of non-labelled cells in 3D.

Besides the options for investigating statistically relevant cell numbers and application in high-throughput analysis, our platform allows for detailed looks on single cell behavior, relevant for cell behavior in spatially heterogeneous microenvironments or studies on cell-cell interactions. As shown in Fig. 3D(i), single cell trajectories can be visualized in a spatio-temporal manner. Furthermore, automatically determined cell parameters, such as cell speed (Fig. 3D(ii)) and morphology (e.g. aspect ratio and roundness) and their changes over time (Fig. 3D(ii)), can be used for a detailed analysis of migratory pattern of cells. To demonstrate the accuracy of automatically determined morphological parameters (aspect ratio, roundness, cell area), results from automatic detection of 161 macrophages were compared with manual measurements using ImageJ (Fig. S5). We could show that morphological parameters were precisely determined (Pearson correlation coefficient: r = 0.79, 0.90 and 0.95 for aspect ratio, roundness and cell area, respectively). Similarly, we demonstrated the accuracy of detected morphological parameters of 127 elongated MDA-MB-231 cells with Pearson correlation coefficient r = 0.91, 0.96 and 0.89 for aspect ratio, roundness and cell area, respectively (Fig. S6).

Overall, the example shows that the developed computation framework is a robust method to track a large number of primary human cells (e.g. macrophages) in 3D biomimetic environments with high local and temporal resolution over long-time periods of several days.

### Concluding remarks

In this work, we present a method for automatic and efficient segmentation and tracking of label-free cells in 3D biomimetic matrices using bright-field time lapse microscopy. The validation results showed that our method is efficient to track and analyze cell migration of primary non-labelled cells at single cell level over time periods of several days. The newly developed 3D single cell tracking technology was demonstrated to be applicable for *in vitro* 3D cell culture studies on the influence of cell-matrix interactions, paracrine cell-cell signals as well as drug screening. Based on the analysis of cell morphology, migration trajectory and genealogy it is possible to correlated the cell response to matrix properties, like pore size, stiffness and the presence of other ECM components, or to gradient cytokine signals from neighboring cells as demonstrated in other reports^36–38^.

Comparing with the state of the art, our approach comprises new features in the cell detection method, allowing automated cell detection without a complex cell library of 3D shapes. Besides that, the method provides insight into dynamic changes of cell morphology, which can be correlated to the cell migratory parameters.

Depending on the scientific question, various cell characteristics can be further analyzed manually or using an external analysis software from the obtained single cell trajectories including genealogies of dividing cells^36^, and cell behavior in complex bioengineered 3D systems e.g. biomimetic matrices with defined biophysical properties^8^ or functionalized with other ECM components^39,40^, short-range cytokine gradients^38,41^ and tissue boundaries^9^ allowing for a better understanding of cell-cell-interactions and mimicking *in vivo* like microenvironments.

## Materials and Methods

### Reconstruction of 3D collagen I matrices

3D Coll I matrices were reconstructed on poly(styrene-alt-maleic anhydride) (PSMA) (Sigma-Aldrich, Germany) coated 13 mm coverslips (Diagonal, Germany), resulting in covalently binding of collagen to PSMA-layer^42^. Type I rat-tail collagen solution (Corning, New York, USA) was diluted with 250 mM phosphate buffered at pH 7.5 (Sigma-Aldrich) to achieve 2 mg/ml Coll I concentration, as described in^8^. Subsequently, prepared Coll I solution were transferred onto the surface of PSMA-coated coverslips and were polymerized for 90 min 37 °C and 95% humidity and 5% CO_2_ content. Reconstituted Coll I matrices were washed twice with PBS and kept in PBS prior to use.

### Fluorescently labelling of cells

1 × 10^6^ human primary macrophages and MDA-MB-231 breast cancer cells were kept in 1 ml RPMI-1640-medium without phenol red (for macrophages; Biochrom, Berlin, Germany) or DMEM (for MDA-MB-231 cells; Biochrom) supplemented with 10% fetal calf serum (Biochrom, Germany) and 1% ZellShield (anti-biotic; Biochrom). Fluorescent labelling solutions, Vybrant® DiD (1:200 dilution; Invitrogen, Germany), 5 µM CellTracker™ Red CMTPX (Invitrogen), 1 µM Hoechst 33342 (Sigma-Aldrich), were separately added to cell suspension, and were incubated at 37 °C, 95% humidity and 5% CO_2_ content for 30 min. Subsequently, cells were washed 3 times with Hank’s Balanced Salt Solution (HBSS; Biochrom).

### Cell culture on 3D Coll I matrices

1 × 10^4^ human primary macrophages or breast cancer cells (MDA-MB-231 cell line) were prepared in RPMI-1640-medium without phenol red (for macrophages) or DMEM (for MDA-MB-231 cells) supplemented with 10% fetal calf serum and 1% ZellShield. For macrophage preparation heparinized blood of human healthy volunteers after informed signed consent (taken in accordance to the regulations and guidelines approved by the ethical committee of the university clinics Leipzig) was purchased from the Institute of Transfusion Medicine at Universität Leipzig and differentiated into macrophages as described elsewhere^35^. Cells were seeded onto prepared 3D Coll I matrices and culture in 24-wells plate at 37 °C, 95% humidity, 5% CO_2_ content for 2 h prior to perform live cell imaging, allowing initial cell adhesion to 3D Coll I matrices.

To assess the phototoxicity induced by time-lapse microscopy, the same number of cells was seeded onto 3D Coll I matrices and cultivated at similar condition of 37 °C, 95% humidity, 5% CO_2_ content in the cell culture incubator.

### Time-lapse experiment (Live cell imaging)

Cell culture plates were placed on Axio Observer Z1 microscope (Zeiss, Jena, Germany) equipped with incubation chamber and motorized stage. The incubation chamber was adjusted to cell culture condition at 37 °C, 95% humidity and 5% CO_2_ content. Sequential images as well as multi-positioning were enabled using a 10 × objective (Zeiss) and Optovar 1.6× module (Zeiss) in bright-field mode by Zen 2012 Software (Zeiss). The acquisition time was set to 50 ms with a LED low turn-on voltage of 2.9 V. Camera settings were identically set for all experiments and illumination was constant throughout the whole time period. Camera settings were identically set for all experiments. Stacked images were gathered every 5 µm in z-direction (625 µm in total) at a 10 min time interval for a total duration of 68 h in bright-field mode. Images were 1388 × 1040 pixels in resolution with a x- and y-voxel size of 0.87 µm.

### Determination of cell viability (WST-1 Assay)

Cell toxicity was performed after finishing the time lapse experiment using cell proliferation reagent WST-1 (Cayman, Germany) and compared with cells cultivated in the cell culture incubator. WST-1 solution was diluted (dilute ratio 1:10) with RMPI-1640 medium without phenol red supplemented with 10% fetal calf serum and 1% ZellShield. Cell culture medium was aspirated and 300 µl of the prepared WST-1 solution were subsequently added. Cells were incubated with prepared WST-1 solution for 30 min at 37 °C, 95% humidity, 5% CO_2_ content in the cell culture incubator. Afterwards, 100 µl of incubated WST-1 solution were transferred to 96-wells transparent plate (Greiner, Germany). An absorption measurement was performed at wavelength  450 nm using 96-wells plate reader (Tecan F200; Tecan, Austria). To compare the cell viability of the different settings, absorption values of cells cultivated in cell culture incubator without treatment were set to 100%.

### Cell tracking software

The 3D single cell tracking platform was written in C++ programming language using the Qt library (The Qt company, Espoo, Finland). The developed stand-alone software follows the model-view-controller design pattern and consists of 3 functional parts, which are responsible for data management, cell detection and cell tracking (Figs S1 and S2).

### Data availability

The software and, datasets generated and analyzed during the current study are available from the corresponding author upon reasonable request.

## Electronic supplementary material

Supplementary Information

Supplementary Video 1

Supplementary Video 2

## References

[1] Friedl P, Gilmour D (2009). Collective cell migration in morphogenesis, regeneration and cancer. Nat. Rev. Mol. Cell Biol..

[2] Even-Ram S, Yamada KM (2005). Cell migration in 3D matrix. Curr. Opin. Cell Biol..

[3] Danen EHJ, Sonnenberg A (2003). Integrins in regulation of tissue development and function. J. Pathol..

[4] Knox P, Crooks S, Rimmer CS (1986). Role of fibronectin in the migration of fibroblasts into plasma clots. J. Cell Biol..

[5] Lacout C (2003). A defect in hematopoietic stem cell migration explains the nonrandom X-chromosome inactivation in carriers of Wiskott-Aldrich syndrome. Blood.

[6] Schroeder T (2011). Long-term single-cell imaging of mammalian stem cells. Nat. Methods.

[7] Friedl P, Alexander S (2011). Cancer invasion and the microenvironment: Plasticity and reciprocity. Cell.

[8] Sapudom J (2015). The phenotype of cancer cell invasion controlled by fibril diameter and pore size of 3D collagen networks. Biomaterials.

[9] Sapudom J, Rubner S, Martin S, Pompe T (2016). Mimicking Tissue Boundaries by Sharp Multiparameter Matrix Interfaces. Adv. Healthc. Mater..

[10] Wolf K (2009). Collagen-based cell migration models *in vitro* and *in vivo*. Semin. Cell Dev. Biol..

[11] Infanger DW, Lynch ME, Fischbach C (2013). Engineered culture models for studies of tumor-microenvironment interactions. Annu. Rev. Biomed. Eng..

[12] Baker BM, Chen CS (2012). Deconstructing the third dimension - how 3D culture microenvironments alter cellular cues. Journal of Cell Science.

[13] Griffith LG, Swartz MA (2006). Capturing complex 3D tissue physiology *in vitro*. Nat. Rev. Mol. cell Biol..

[14] Yamada KM, Cukierman E (2007). Modeling Tissue Morphogenesis and Cancer in 3D. Cell.

[15] Bray LJ (2015). Multi-parametric hydrogels support 3D invitro bioengineered microenvironment models of tumour angiogenesis. Biomaterials.

[16] Stamov DR, Pompe T (2012). Structure and function of ECM-inspired composite collagen type I scaffolds. Soft Matter.

[17] Atala A, Kasper FK, Mikos AG (2012). Engineering complex tissues. Sci. Transl. Med..

[18] Franke K, Sapudom J, Kalbitzer L, Anderegg U, Pompe T (2014). Topologically defined composites of collagen types i and v as *in vitro* cell culture scaffolds. Acta Biomater..

[19] Frigault MM, Lacoste J, Swift JL, Brown CM (2009). Live-cell microscopy - tips and tools. J. Cell Sci..

[20] House, D., Walker, M. L., Wu, Z., Wong, J. Y. & Betke, M. Tracking of cell populations to understand their spatio-temporal behavior in response to physical stimuli. In *2009 IEEE Conference on Computer Vision and Pattern Recognition*, *CVPR 2009* 186–193, 10.1109/CVPR.2009.5204057 (IEEE, 2009).

[21] Rapoport DH, Becker T, Mamlouk AM, Schicktanz S, Kruse C (2011). A novel validation algorithm allows for automated cell tracking and the extraction of biologically meaningful parameters. PLoS One.

[22] Progatzky F, Dallman MJ, Lo Celso C (2013). From seeing to believing: labelling strategies for *in vivo* cell-tracking experiments. Interface Focus.

[23] Coutu DL, Schroeder T (2013). Probing cellular processes by long-term live imaging–historic problems and current solutions. J. Cell Sci..

[24] Pattison, D. I. & Davies, M. J. In *Exs* 131–157, 10.1007/3-7643-7378-4_6 (Birkhäuser-Verlag, 2006).

[25] Dixit R, Cyr R (2003). Cell damage and reactive oxygen species production induced by fluorescence microscopy: effect on mitosis and guidelines for non-invasive fluorescence microscopy. Plant J..

[26] Eilken HM, Nishikawa S-I, Schroeder T (2009). Continuous single-cell imaging of blood generation from haemogenic endothelium. Nature.

[27] Meijering E, Dzyubachyk O, Smal I (2012). Methods for cell and particle tracking. Methods Enzymol..

[28] Awasthi V, Doolittle KW, Parulkar G, McNally JG (1994). Cell tracking using a distributed algorithm for 3-D image segmentation. Bioimaging.

[29] Adanja I, Megalizzi V, Debeir O, Decaestecker C (2011). A new method to address unmet needs for extracting individual cell migration features from a large number of cells embedded in 3D volumes. PLoS One.

[30] Metzner C (2015). Superstatistical analysis and modelling of heterogeneous random walks. Nat. Commun..

[31] Horan PK, Slezak SE (1989). Stable cell membrane labelling. Nature.

[32] Purschke M, Rubio N, Held KD, Redmond RW (2010). Phototoxicity of Hoechst 33342 in time-lapse fluorescence microscopy. Photochem. Photobiol. Sci..

[33] Parish CR (1999). Fluorescent dyes for lymphocyte migration and proliferation studies. Immunol. Cell Biol..

[34] Mazzaferri J, Roy J, Lefrancois S, Costantino S (2015). Adaptive settings for the nearest-neighbor particle tracking algorithm. Bioinformatics.

[35] Friedemann M (2017). Instructing Human Macrophage Polarization by Stiffness and Glycosaminoglycan Functionalization in 3D Collagen Networks. Adv. Healthc. Mater..

[36] Scherf N (2012). On the symmetry of siblings: Automated single-cell tracking to quantify the behavior of hematopoietic stem cells in a biomimetic setup. Exp. Hematol..

[37] Sapudom J, Rubner S, Martin S, Pompe T (2016). Tissue Boundaries: Mimicking Tissue Boundaries by Sharp Multiparameter Matrix Interfaces (Adv. Healthcare Mater. 15/2016). Adv. Healthc. Mater..

[38] Ansorge M (2016). Short-range cytokine gradients to mimic paracrine cell interactions *in vitro*. J. Control. Release.

[39] Sapudom J (2015). The interplay of fibronectin functionalization and TGF-β1 presence on fibroblast proliferation, differentiation and migration in 3D matrices. Biomater. Sci..

[40] Sapudom J (2017). Molecular weight specific impact of soluble and immobilized hyaluronan on CD44 expressing melanoma cells in 3D collagen matrices. Acta Biomater..

[41] Ansorge M (2017). Mimicking Paracrine TGFβ1 Signals during Myofibroblast Differentiation in 3D Collagen Networks. Sci. Rep..

[42] Pompe T (2003). Maleic anhydride copolymers - A versatile platform for molecular biosurface engineering. Biomacromolecules.

