# Supplementary material for: Quantitative label-free single cell tracking in 3D biomimetic matrices
Source: Sci Rep. 2017 Oct 26;7:14135. doi: 10.1038/s41598-017-14458-x (PMC5658366; doi:10.1038/s41598-017-14458-x)
Supplement: Supplementary file 1 — Supplementary Information [file 41598_2017_14458_MOESM1_ESM.pdf]

Supplementary information

Quantitative label-free single cell tracking in 3D biomimetic matrices

Jiranuwat Sapudom [a,§]
Johannes Waschke [a,b,§]
Katja Franke [a]
Mario Hlawitschka [c]
Tilo Pompe [a,*]

[a] Institute of Biochemistry, Faculty of Biosciences, Pharmacy and Psychology, Universität Leipzig, Leipzig 04103, Germany.
[b] Institute of Computer Science, Faculty of Mathematics and Computer Science, Universität Leipzig, Leipzig 04103, Germany.
[c] Professorship for Computer Graphics, Faculty of Computer Science, Mathematics and Natural Science, Hochschule für Technik, Wirtschaft und Kultur Leipzig, 04277 Leipzig, Germany

[§] equally contributing authors

Correspondence: Prof. Dr. Tilo Pompe**;**
Institute of Biochemistry, Faculty of Biosciences, Pharmacy and Psychology, Universität Leipzig, Leipzig 04103, Germany.
Telephone/Fax: +49 341 97 36931/9
Email: tilo.pompe@uni-leipzig.de

Keywords:

cell migration, label-free detection, bright-field microscopy, single cell analysis, 3D microenvironment

Cell Tracking Software

We developed a software tool that covers the full chain of steps necessary to detect and track cells. Software screen shot (Figure S1) and class scheme (Figure S2) are depicted. The software receives a 2D or 3D microscope video, which must be provided as a stack of image files. If the data contain only moving cells, background subtraction can be applied to remove fixed image parts and thus to reduce artifacts. However, slowly moving - or dead - cells could disappear in such a processing step. To ensure unbiased results it is recommended to disable background subtraction in such cases.

The next step in the workflow is the cell detection configuration. The software provides a configuration view that shows a preview of one time step on the basis of the currently selected cell detection parameters. Typical parameters are search radii, denoising values, sensitivity thresholds and the selection of a reference template for both cell and background region. It is also possible to extend the software and to select between different detection algorithms.

After cell detection, it is possible to adjust the results manually (changing, deleting and creating cell objects with the cursor) and to post-process cell shapes with automatic tools (see section 'Correction of detected cell shape' in main text).

A cell set for each time step is stored. In a consecutive step, the tracking algorithm calculates temporal relations of the cell objects. It is again possible to set up parameters and verify them in a preview window. The software provides post-processing options for tracks as well. Common problems, like early stopping tracks, can be connected automatically on basis of distance metrics. In addition, fully manual processing options are available as well. The final results, including trajectory and temporal changes of cell shape parameters, can be exported to csv tables and R plots.

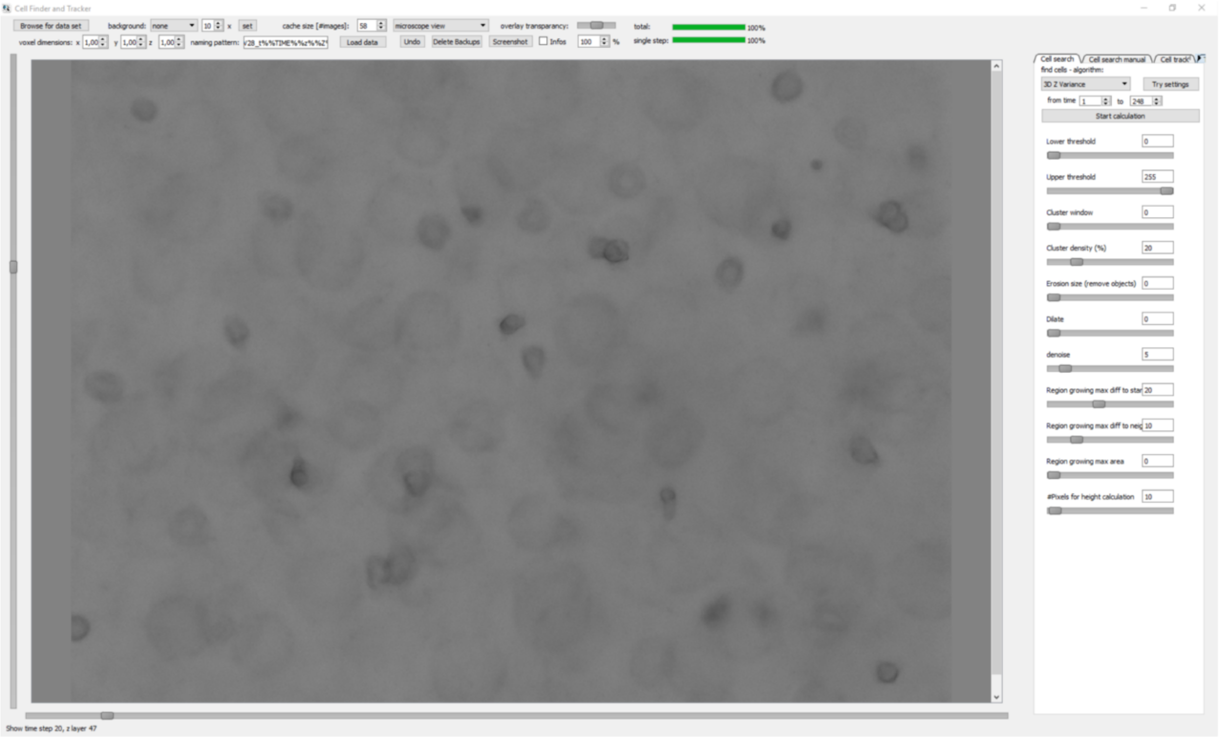

Figure S1

Screen shot of cell tracking software.

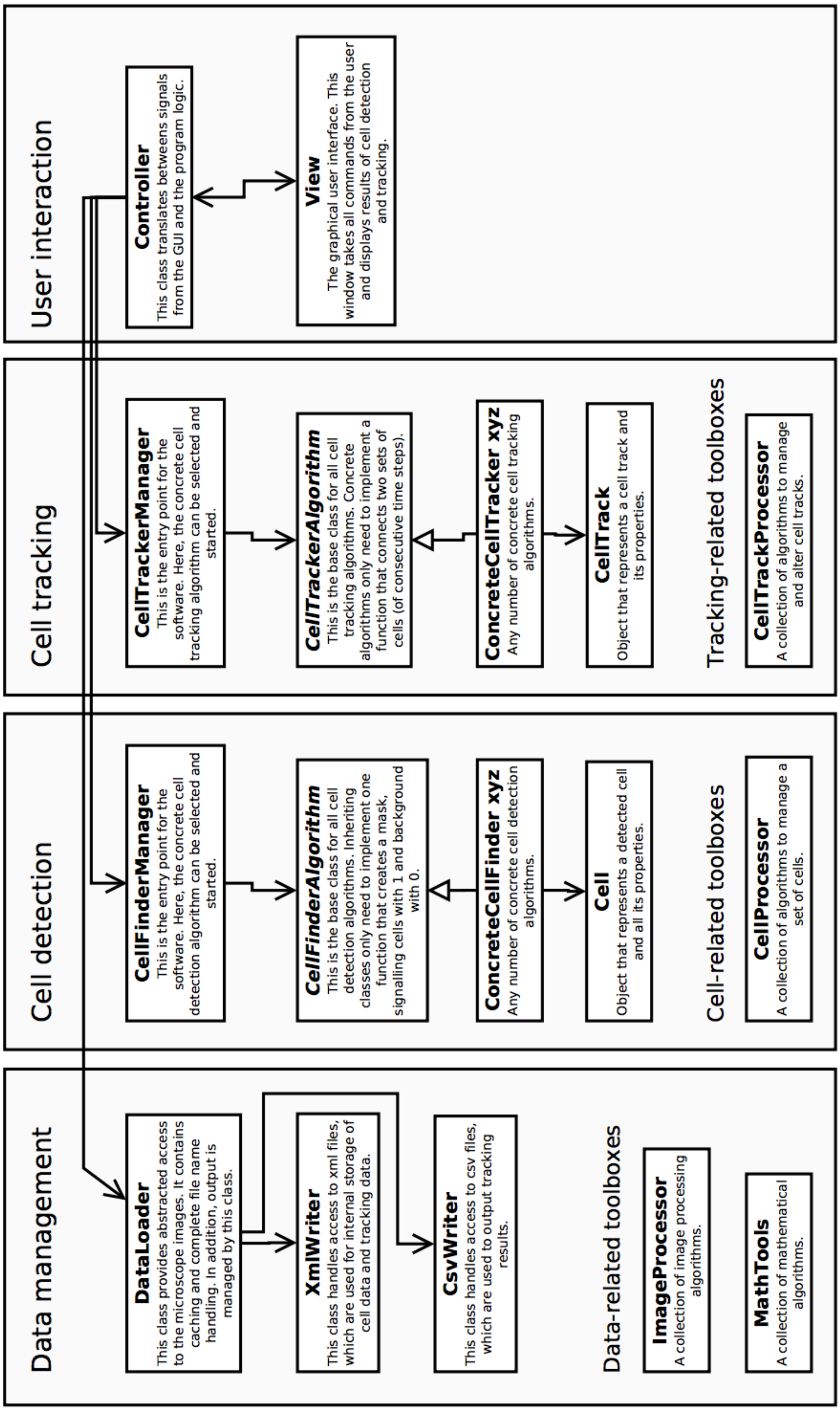

Figure S2.

Class scheme of tracking software.

Technical details

Template definition

The sampling process starts with the selection of a position of interest and its n closest voxels inside the xy-plane. For each of the selected voxels, the height profile along the z-axis within a user-defined distance r is retrieved, and, finally, an averaged z-profile t for this cell is calculated. The distance should be chosen big enough to span the characteristic refraction signal.

$$t = \frac{1}{n}\sum_{i=1}^{n}\begin{pmatrix} p(x_i, y_i, z-r) \\ p(x_i, y_i, z-r+1) \\ p(x_i, y_i, z-r+2) \\ \dots \\ p(x_i, y_i, z+r) \end{pmatrix}$$

$p(x, y, z)$	intensity at coordinates x, y, z
x_i, y_i	x and y coordinates of the ith voxel, where larger i name voxels that are more distant from the position of interest

3D cell detection

The z-profile is compared (using Pearson correlation) to the *cell template* as well as to the *background reference*. If correlation to the *cell template* is higher than correlation to the *background template* the current xyz-position is marked with a digital marker as part of a cell.

The template matching process results in a 3D cell map that contains labels for estimated cell regions. The 3D cell map contains not only distinctive clusters of cells, but scattered small regions of false positives, which are caused by noise or the existence of small objects that have similar refraction influence on the z-intensity profile as a *cell template*. To reduce those artifacts, subsequently a cluster algorithm was used. For each position of the cell map, a neighborhood of a certain radius is considered. If the percentage of marked voxels exceeds a pre-defined threshold (e.g. 20% of a 3×3×3 window), the specific position is marked as cell inside a second cell map. Finally, the second cell map contains only cell labels at xyz-positions that had been densely labeled in the neighborhood during the original template matching process.

The cell map now contains two classes of voxels: background regions and voxels marked as cells. In a following step, connected parts of the cell map are extracted. Due to characteristics of the bright-field images and the template matching process, cells are not only

detected at their correct z-position but also at neighboring z-stack positions. This results in a strongly overestimated cell volume. A first step to overcome this problem is to flatten the cell by deleting all voxels that are out of a respective z-stack layer which has the largest area. After this process, cells are flat objects that are situated at a rough preliminary z-position. In a consecutive step, the precise z-position is calculated (see main text Results and discussion in section 3.2.2) and shape post-processing is applied (see main text Results and discussion in section 3.2.3).

Cell shape parameters

Cells are internally stored as a list of n voxel coordinates $v = \{v_1, v_2, \dots, v_n\}$. Since in our software cells are usually considered as a flat object (a volume calculation would be inaccurate due to the low z resolution), all voxel coordinates of one cell have the same z-position.

Cell centroid

The centroid of a cell is used as the average position of a cell.

$$f_{centroid} = \sum_{i=1}^{n} \frac{v_i}{n}$$

Cell area

We store each voxel in which we assume the cell is located. Therefore, the total cell area is calculated as the product of the number of voxels n and the *area* of a single voxel. The area of a single voxel depends on the microscope resolution and output image size.

$$f_{area} = n * area$$

Major and minor axes and their lengths (Cell orientation)

These values describe the largest and smallest cuts that can be made through an object. As constraint, only cuts are allowed that pass the cell centroid (otherwise the minimum diameter could be erroneously assumed as any small cut along the border region). We calculate this numerically by testing cuts across the cell centroid in e.g. 180 different rotations. The longest and the shortest cuts are taken as major and minor axes and their respective lengths are stored as d_{max} and d_{min}. The angle of the major axis is used to describe the cell orientation.

Aspect ratio

Aspect ratio describes the ratio of major and minor axes length:

$$f_{aspect} = d_{max}/d_{min}$$

Cell roundness

Roundness is calculated as follows, indicating deviations from ideal circular objects with parameter 1 being a circle and lower values down to 0 being cell shapes with non-circular elongated or fingering shapes:

$$f_{roun\,dness} = \frac{4 * f_{area}}{d_{max}^2 * \pi}$$

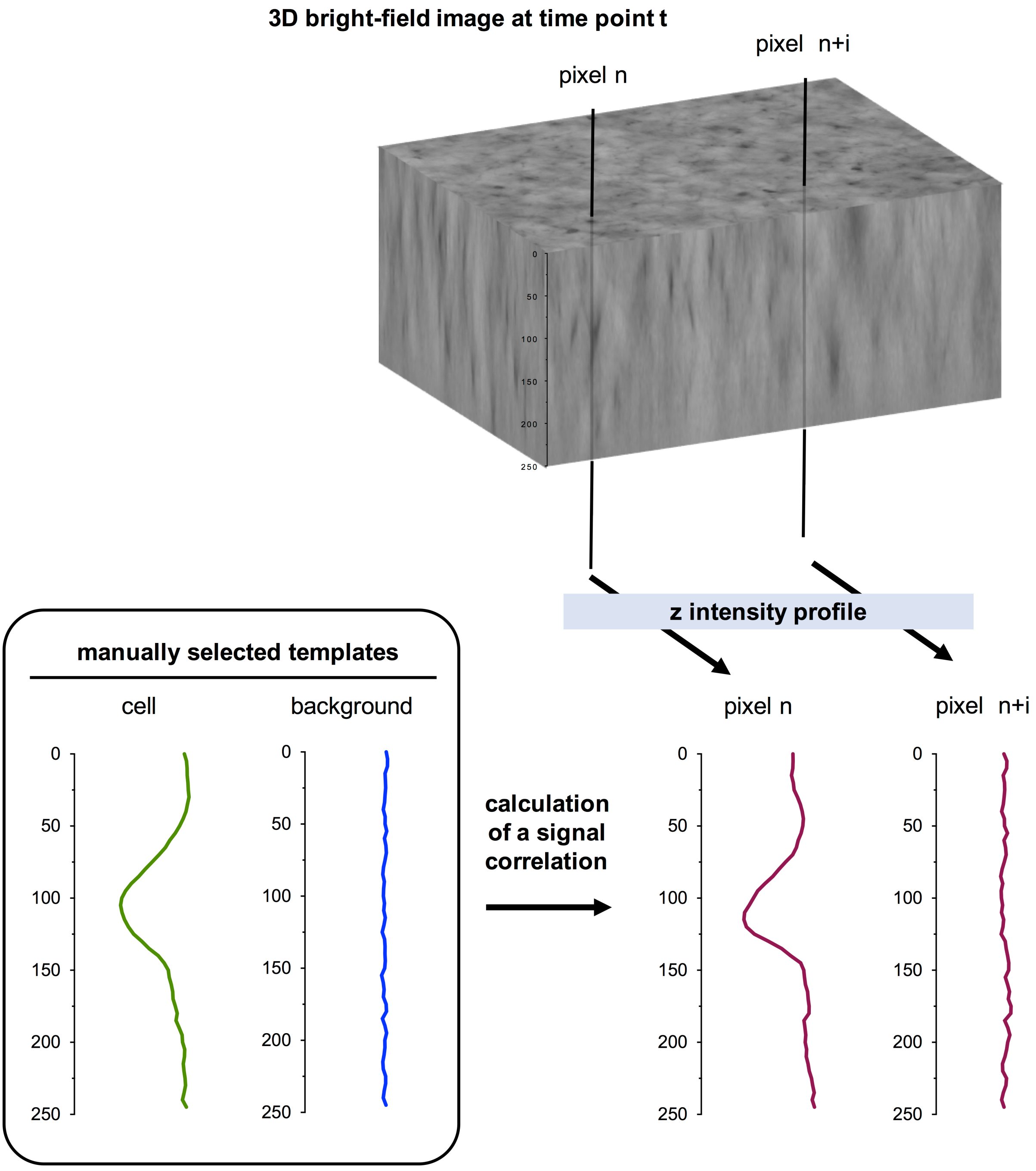

Figure S3.

Cell detection using 1D templates. The intensity profile along the z-axis is generated for both cell and background (Coll I matrices) voxels as a function of z-distance, and was set as a 1D template. The correlation between 1D template and z-intensity profile at each xyz-position position was calculated.

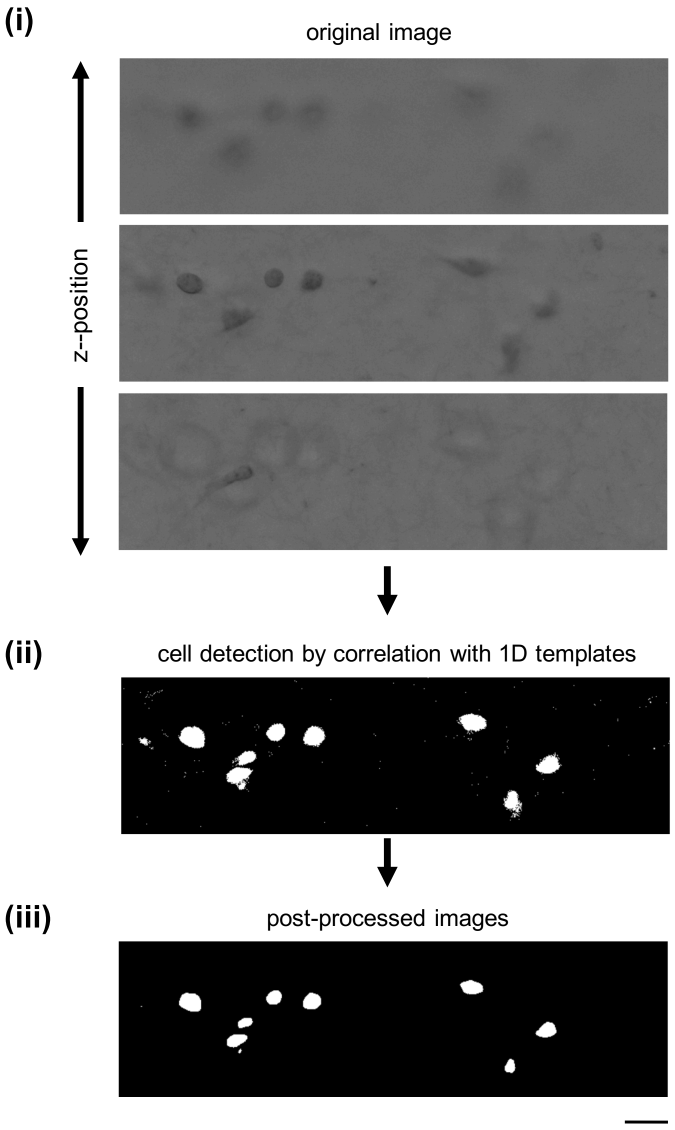

Figure S4.

Example of cell detection work flow. (i) Original image stack with different z-positions, (ii) results from cell detection using 1D templates and (iii) detected cells after elimination of noise. (Scale bar = 100 μm)

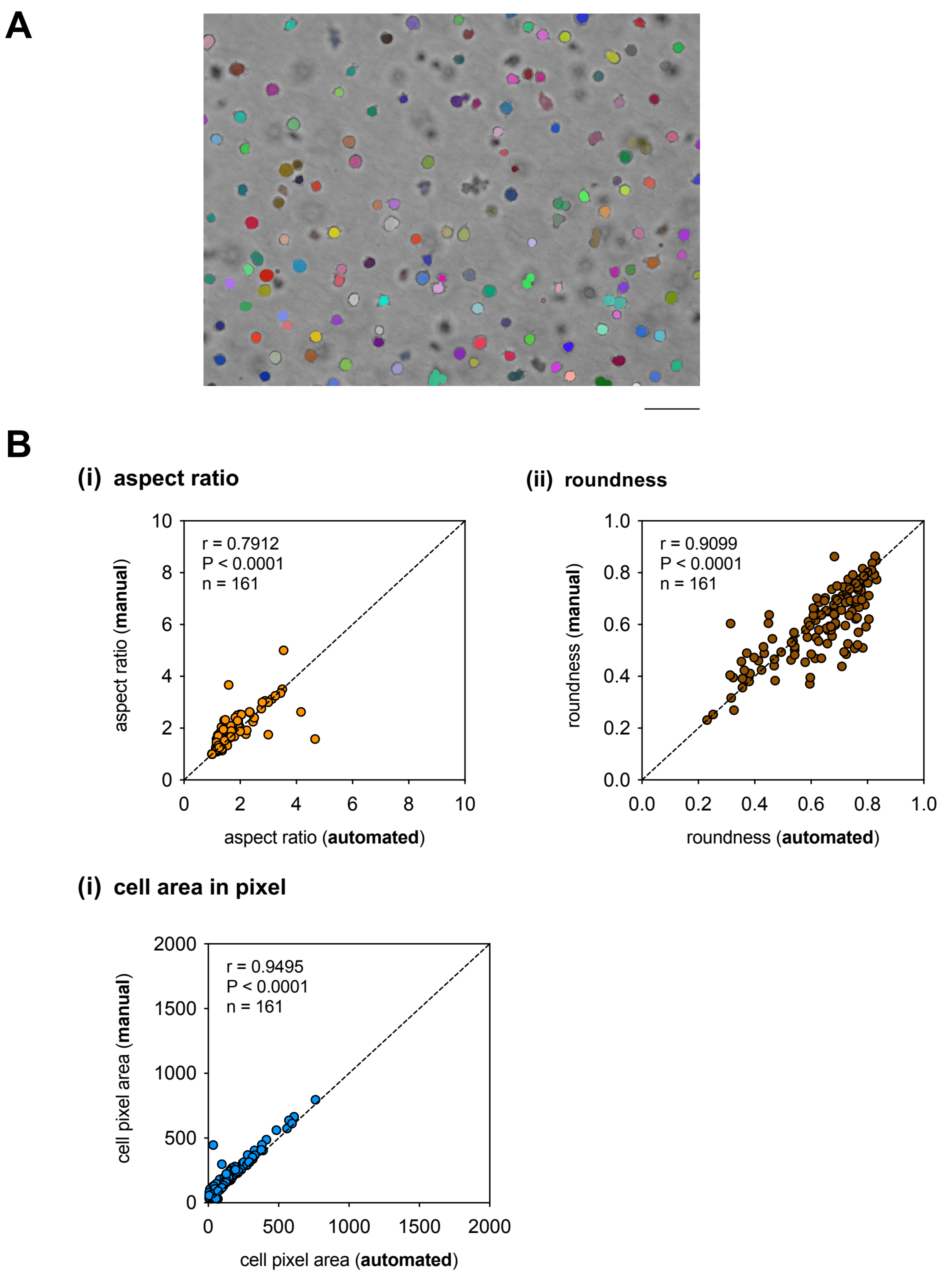

Figure S5.

Accuracy of morphological analysis of macrophages in 3D Coll I matrices. (A) An example of the result from morphological detection. (Scale bar = 200 µm) (B) Morphological parameters (i) aspect ratio, (ii) roundness and (iii) cell area of macrophages are compared between manual measurements using ImageJ and the automated detection using the developed computation framework. 161 macrophages were analyzed. Pearson correlation was used for comparing manual and automated determination of cell morphological parameters.

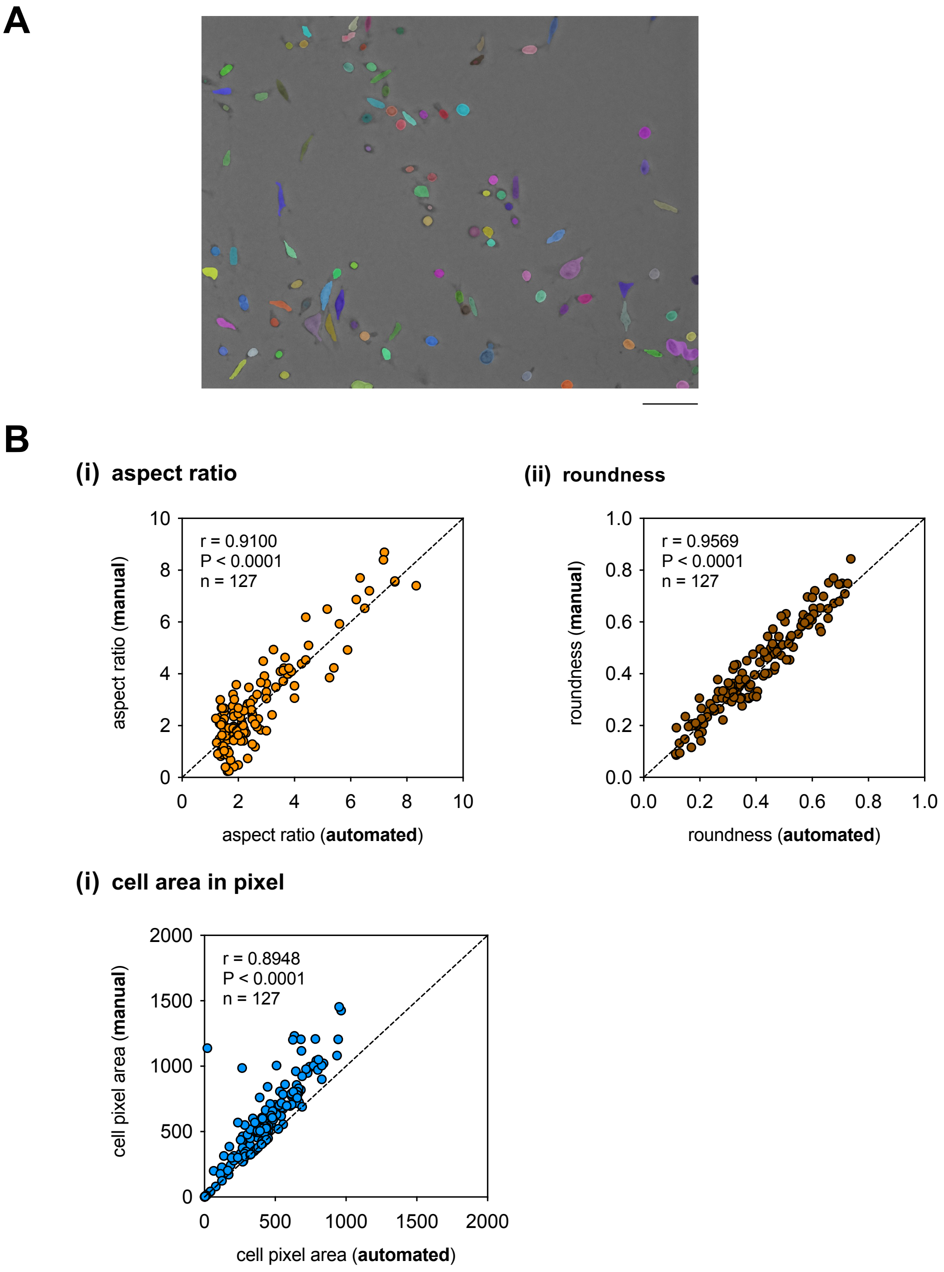

Figure S6.

Accuracy of morphological analysis of MDA-MB-231 in 3D Coll I matrices. (A) An example of the result from morphological detection. (Scale bar = 200 μm) (B) Morphological parameters (i) aspect ratio, (ii) roundness and (iii) cell area of MDA-MB-231 are compared between manual measurements using ImageJ and the automated detection using the developed computation framework. 127 cells were analyzed. Pearson correlation was use for comparing manual and automated determination of cell morphological parameters.

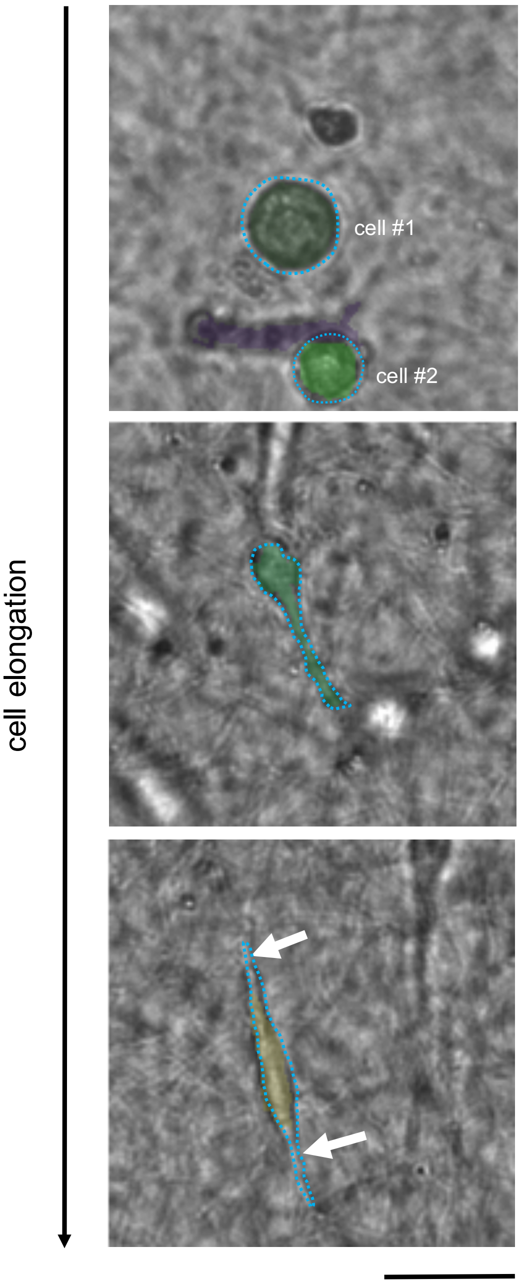

cell #1

properties	automated detection	manual detection
area (pixel)	2053	2280
aspect ratio	1.102	1.076
roundness	0.908	0.930

cell #2

properties	automated detection	manual detection
area (pixel)	1151	1356
aspect ratio	1.212	1.114
roundness	0.825	0.906

properties	automated detection	manual detection
area (pixel)	1264	1684
aspect ratio	6.513	6.492
roundness	0.154	0.154

properties	automated detection	manual detection
area (pixel)	1475	1817
aspect ratio	4.646	8.275
roundness	0.215	0.121

Figure S7.

Morphology detection of elongated MDA-MB-231 cells using the develop algorithm. Cell morphological parameters (cell area, aspect ratio and roundness) were compared between manual measurements using ImageJ and the automated detection using the developed computation framework as a function of cell elongation. White arrow showed the limits of the automated cell shape detection to detect very thin cell filopodia similar in size to collagen fibrils of the 3D scaffold. (Scale bar = 50 µm)

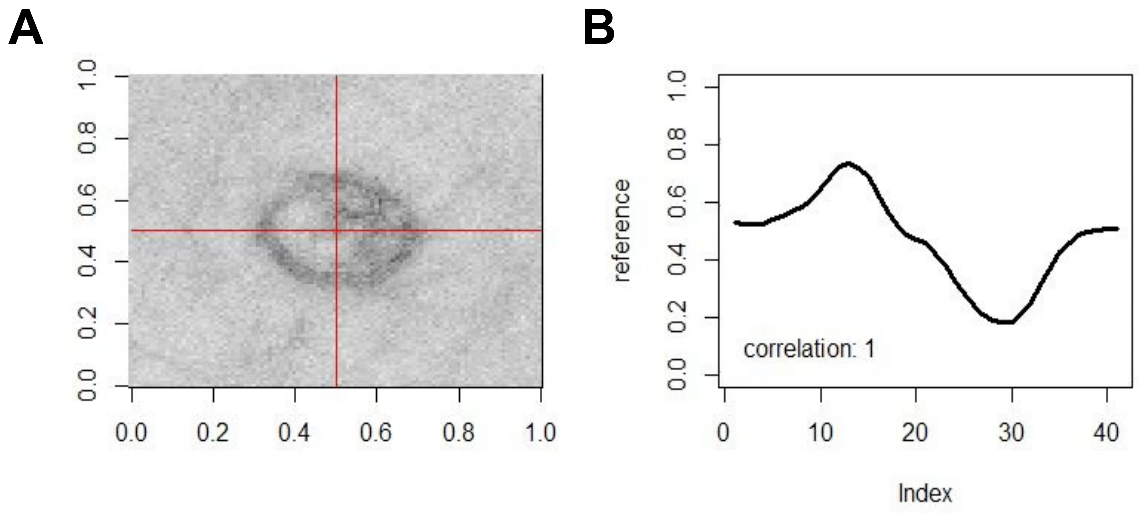

Supplement video 1.

Correction of detected cell shape with region growing, using the z intensity profile similarity as stopping criterion. (A) Bright-field image and (B) z-intensity signal at marked position in A (red cross) and the calculated correlation coefficient are shown.

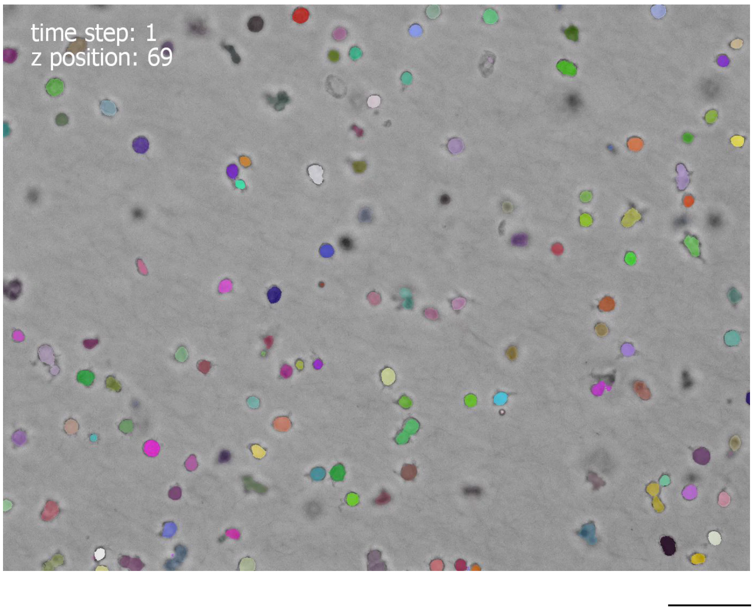

Supplement video 2.

Analyzed dataset using the developed techniques without any manual correction. The data used in this experiment contained 125 z-stacked images (1388 × 1040 pixels; 5 µm z-intervals) and 408 time steps (image acquisition at 10 min intervals for 68 h), which correspond to a total of 51000 images. The image shows only cells, tracks and morphological parameters at z-position #69. (Scale bar = 200 µm)
